# Isothermal inactivation of *Mycobacterium avium* subsp. *paratuberculosis* in curd simulating the stretching phase in pasta-filata cheese process

**DOI:** 10.3389/fmicb.2022.1052222

**Published:** 2022-12-01

**Authors:** Filippo Barsi, Elena Dalzini, Simone Russo, Elena Cosciani-Cunico, Paola Monastero, Norma Arrigoni, Chiara Anna Garbarino, Claudia Cortimiglia, Marina Nadia Losio, Matteo Ricchi

**Affiliations:** ^1^National Reference Centre and WOAH Reference Laboratory for Paratuberculosis, Istituto Zooprofilattico Sperimentale della Lombardia e dell'Emilia Romagna “Bruno Ubertini”, Piacenza, Italy; ^2^National Reference Centre for Emerging Risks in Food Safety, Istituto Zooprofilattico Sperimentale della Lombardia e dell'Emilia Romagna “Bruno Ubertini”, Milan, Italy; ^3^Food Control Division, Istituto Zooprofilattico Sperimentale della Lombardia e dell'Emilia Romagna “Bruno Ubertini”, Brescia, Italy

**Keywords:** thermal resistance kinetics, *Mycobacterium* spp., pasta-filata cheese, food safety, inactivation models, paratuberculosis

## Abstract

Raw milk and dairy products are usually considered the major sources of *Mycobacterium avium* subsp. *paratuberculosis* (MAP) exposure for humans. During the production process of mozzarella cheese, as well as of other pasta-filata cheeses made with pasteurized or raw milk, curd is heated and stretched by addition of hot or boiling water. This step is the critical point for the inactivation of MAP during the production process, but, to our knowledge, no studies have been published about the thermal death time values of MAP in curd. The aim of this study was to determine the inactivation kinetics of MAP in curd used to produce pasta-filata cheese in six independent experiments. The milk was inoculated with a mix of MAP strains (field and registered strains) and, with the aim to simulate the thermal treatment of the curd during the stretching step, samples of 10 g of contaminated curd were vacuum packed and treated separately at six different temperatures from 60°C to 75°C in a water bath. MAP survival was then evaluated by plate count method and inactivation parameters were estimated for determining the thermal resistance of the pathogen directly in the curd. D-values increased from 0.15 min (D_75_-value) to 4.22 min (D_60_-value) and the calculated z-value was 10.2°C. These data aid: (i) to design food thermal process treatments defining acceptance limits of critical control points to ensure safety against MAP; (ii) to predict the time/temperature combinations needed to obtain a certain MAP log reduction during the curd stretching step; (iii) to optimize or validate pasta-filata cheese process.

## Introduction

*Mycobacterium avium* subsp. *paratuberculosis* (MAP) causes paratuberculosis or Johne’s Disease in ruminants, but it is also suspected to be involved in the pathogenic mechanisms of some human disease, like Crohn’s disease (Chiodini et al., 2012), Type I diabetes and others (Scanu et al., 2007; Sisto et al., 2010; Cossu et al., 2011; Dow, 2012; Atreya et al., 2014).

Milk and dairy products are considered among the major sources of MAP-exposure to humans (Gill et al., 2011). Although the direct secretion of MAP into bovine milk by infected animals is considered low (Sweeney et al., 1992; Giese and Ahrens, 2000), fecal contaminations, especially during the milking process, can increase MAP concentration in tank milk (Cocito et al., 1994; Rademaker et al., 2007; Vissers et al., 2007; Atreya et al., 2014). On the other hand, some studies elicited how technical processes and pasteurization can dramatically reduce the MAP load originally present in milk into final dairy products (Eltholth et al., 2009; Gill et al., 2011; Okura et al., 2012). Indeed, many surveys carried out in different types of cheese in different parts of the world, underlined the presence of viable MAP (Williams and Withers, 2010), MAP DNA (Clark et al., 2006; Stephan et al., 2007; Botsaris et al., 2010) or both (Ikonomopoulos et al., 2005; Faria et al., 2014; Galiero et al., 2016), showing that the survival of MAP to the manufacturing processes and ripening phases is possible, but a great variability during cheesemaking occurs. As reported in a review by Gill et al. (2011), three challenging studies on the survival of MAP during the production of cheeses made by distinctive starter cultures and different kind of milk used (sheep, goat and cow or mixtures in different proportions; Sung and Collins, 2000; Spahr and Schafroth, 2001; Donaghy et al., 2004), underlined how different technological processes and ripening times can have a different impact on MAP survival.

Mozzarella cheese, as well as pasta-filata cheeses are typical Italian cheeses made by bovine or buffalo raw or pasteurized milk. Notably, the use of pasteurized milk is generally indicated for this kind of cheese, but the use of fresh raw milk is also allowed. The cheese production is characterized by curd making followed by a heat treatment during the curd stretching by addition of hot or boiling water. This step is critical for the MAP survival during the cheesemaking, but, to our knowledge, no studies have been published about the thermal death time values of MAP in curd.

Mathematical models represent important tools for describing and predicting the growth, survival or inactivation responses of pathogens under specific environmental conditions. During thermal process, predictive kinetic models allow to calculate combinations of times and temperatures necessary to calculate the reduction rate in the bacterial load. This value can be estimated by process lethality (F), which is usually described as the time required to cause log decrease in bacterial numbers at a given reference temperature (Murphy et al., 2002). Other very important parameters are D- and z-values, which are dependent by microorganism and food product. The simulation of production process can be useful to set up tests to estimate the reduction of pathogen concentrations during heat treatments typical of production processes, avoiding the intentional introduction of dangerous microorganisms into the processing environment.

Here, the aim of this study was to determine the inactivation kinetics of MAP in curd used to produce pasta-filata cheese. The determination of thermal death time is pivotal for the knowledge of MAP survival in this type of cheeses and may be used to enhance the microbiological safety of the product.

## Materials and methods

### Experimental design

Six independent experiments were performed in this study. In each trial, 1 l of milk was inoculated with a mix of MAP strains (field isolates and reference strain ATCC 19698) and 2 l with physiological solution (control milk). The curd was manufactured in the pilot plant at the IZSLER’s laboratories. Control samples were then analyzed to evaluate pH, water activity (aw) and Lactic acid bacteria concentration. Then, in order to simulate the thermal treatment of the curd during the stretching step, samples of 10 g of contaminated curd were vacuum packed and treated separately at six different temperatures from 60°C to 75°C in a water bath. MAP survival was then evaluated by plate count method and inactivation parameters were estimated for determining the thermal resistance of the pathogen directly in the curd.

### Bacterial strains and inoculum preparation

A multi-strain suspension of MAP, all type C isolated from feces, including bovine reference strain ATCC 19698 (Hsu et al., 2011), field strains MAP 91/2016 (from bovine) and MAP 187/2011 (from buffalo) was used to contaminate the pasteurized milk during the experiments. The field strains belonged to the National Reference Centre for Paratuberculosis’s collection (IZSLER, Piacenza, Italy). Each strain was cultured as previously described by Cammi et al. (2019). Briefly, the strains were separately cultured in Middlebrook 7H9 broth medium, containing 10% (v/v) Middlebrook OADC Enrichment (Becton Dickinson, United States) and Mycobactin J (2 mg/l; IDvet Innovative Diagnostics, Montpellier, France) for 4–6 weeks at 37°C in a shaker incubator.

At the end of the incubation period, each culture was centrifuged at 2500 ×*g* for 20 min and the pellets were resuspended in: (i) the same volume of growth broth (previously described) supplemented with 5% glycerol to stock the culture at −80°C; and (ii) phosphate buffered saline (PBS). This last suspension was used to calculate MAP concentration of each stock culture. Briefly, decimal dilutions of the suspensions were streaked on Herrold’s egg yolk medium supplemented with Mycobactin (2 mg/l), Nalidixic acid, Vancomycin and Fungizone (HEY ANV) and analysed by f57-qPCR with the genomic equivalent method. The standard for the latter method was obtained from DNA isolated from a lyophilised MAP isolate in the framework of determining some reference material for DNA from MAP (Beinhauerova et al., 2021).

Before the milk contamination, each suspension was thawed at 4°C, centrifuged at 2500 ×*g* for 20 min and the sediment was resuspended in the same volume of PBS. The suspensions were declumped forcing them through a 24 G needle (4 times) and mixed in equal concentration to obtain the multi-strain suspension of MAP.

### Milk contamination and preparation of curd samples

A total of 18 l of fresh pasteurized whole milk (for 100 ml: fat 3.6 g, lactose 4.9 g, protein 3.2 g, salt 0.1 g, Centrale del Latte di Brescia S.p.A., Brescia, Italy) was used in this study. For each experiment, 1 l of milk was inoculated with 1% v/v of multi-strain cocktail of MAP, in order to obtain a concentration of 6 log CFU/mL in milk and to produce contaminated curd, while 2 l of milk were inoculated with 1% v/v of sterile physiological solution to produce control curd. Then, 1% w/v of freeze-dried starter culture (*Streptococcus thermophilus* and *Lactobacillus delbrueckii* subsp. *bulgaricus*; Insao S.r.l., Milan, Italy) and 0.12% v/v of liquid calf rennet (strength 1:18000) were also added to milk. After 60 at 36 ± 2°C, the coagulated milk was cut, gently stirred to separate the whey and placed in perforated molds (25 cm diameter and 18 cm height). The curd remained under the whey and let stand at 32 ± 2°C for 4 h until the mature curd (pH of 5.2 ± 0.1), one contaminated and two not contaminated for each experiment, were obtained.

**Figure 1:**
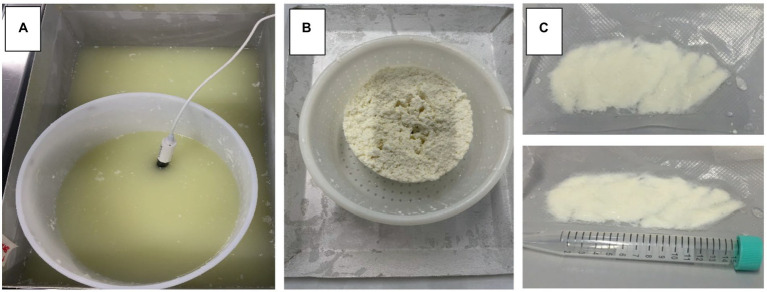
Pictures collected during the experimental trials and representative of curd maturation under whey **(A)**, mature curd **(B)** and vacuum-packed curd samples before the thermal treatment **(C)**.

The contaminated mature curd was cut in small slices using a sterile blade. Portions of 10 g were transferred in polyethylene bags and then pressed to obtain samples with a uniform thickness of *ca.* 5 mm and vacuum packed using S100-Tecnovac equipment (Tecnovac, Bergamo, Italy). Figure 1 illustrates some pictures taken during the curd preparation until the vacuum-packed samples were obtained.

### Isothermal inactivation experiments

The thermal inactivation of MAP in curd was estimated at the temperatures of 60, 64, 68, 70, 73 and 75°C, as independent experiments. Vacuum-packed samples were immersed in circulating water bath (Memmert, EN.CO Srl, Venice, Italy) maintained at the set temperature. The temperatures were monitored and registered using a Thermo Button 22 l data logger (Astori Tecnica s.n.c., Brescia, Italy) placed in a vacuum-packed control sample of curd.

For each experiment, the temperature reached in the curd samples was monitored also in real time in one sample using a digital thermometer (PeakTech^®^ Digital Thermometer 5,135, Ahrensburg, Germany) to determine the start of the experiment, corresponding to the achievement of the target temperature.

For all the thermal experiments, the come-up time (time to reach the set temperature) was not included in the total treatment time with the aim to obtain consistent isothermal treatments. Thus, starting from the time when the curd samples reached the target temperature, samples were removed from the water bath at different time intervals and for different total time (Table 1) depending on the specific target temperature. Upon removal, samples were immediately cooled in an ice-water bath to stop the heat exposure.

**Table 1:** Temperature applied during the isothermal experiment and the respective sampling time.

Temperature (°C)	Sampling time (min)						
60°C	0	2	4	6	8	10	
64°C	0	2	4	6	8	10	
68°C	0	1.5	2.5	3	3.5		
70°C	0	0.5	1	1.5	2		
73°C	0	0.05	0.1	0.15	0.2	0.25	0.3
75°C	0	0.05	0.1	0.15	0.2	0.25	0.3

### Microbiological and physico-chemical analysis

Microbiological analyses were performed on milk through direct plate count. In curd, samples of 10 g were homogenised using 90 ml of cheese diluent made with casitone, 1% (w/v; Becton Dickinson, United States), sodium citrate 2% (w/v; Carlo Erba, Milan, Italy), sodium chloride 0.5% (w/v; VWR Prolabo, Fontenay Sous Bois, France), for 3 min in a Stomacher 400 blender (Seward Medical, London, United Kingdom) and then the suspensions were warmed in water bath at 37°C for 40 min. Samples were homogenised again for 3 min and 1 ml of homogenate was taken and serially tenfold diluted in Buffered Peptone Water (BPW).

For contaminated samples of milk and homogenised cheese, MAP enumeration was performed by plating 1 ml and 0.1 ml of serial 10-fold dilutions in Herrold’s egg yolk medium (HEYM), containing sodium pyruvate (4 g/l) and supplemented with Mycobactin J (2 mg/ml) and Nalidixic acid (50 mg/l), Vancomycin (50 mg/l) and Fungizone (5 mg/l). Duplicate plates were incubated for 3 months at 37°C after tape sealing.

Suspected colonies were confirmed by Ziehl Neelsen staining and f57 PCR (Ricchi et al., 2016).

On control samples (not contaminated), MAP enumeration was performed in milk before all the experiment, to verify the absence of natural contamination. LAB concentration was determined according to ISO 15214 ((ISO), I.O.f.S, 1998) and the pH value was measured using pH meter MU 6100 l (VWR International S.r.l., Milan, Italy) in two replicate samples of milk and curd. The aw value was measured at 25°C with the aw recorder AquaLab, series 3, Model TE (Decagon Devices, Inc., Pullman, WA, United States) according to ISO 18787 ((ISO), I.O.f.S, 2017) in two replicate samples of curd.

### Data analysis

The microbial results were expressed as colony forming unit (CFU) per g and converted to log CFU per g. To obtain the thermal inactivation curves of MAP, the log concentrations were plotted against the time and the inactivation kinetic modeling was performed using Geeraerd log-linear+tail model (Eq. 1; Geeraerd et al., 2000) or log-linear regression (Eq. 2) by GInaFit software, a free add-in for Microsoft^®^ Excel developed by Geeraerd et al. (2005).


(1) $$\log\left(N\right)=\log\left(\left(10^{\log N_{0}}-10^{\log N_{\mathrm{res}}}\right)\right)e^{\left(-k_{\max}t\right)}+10^{{\mathrm{logN}}_{\mathrm{res}}}$$

(2) $$\log\left(N\right)=\log\left(N_{0}\right)-\frac{K_{\max}*t}{\ln\left(10\right)}$$

where: N is the microbial concentration (CFU/g), N_0_ is the initial microbial concentration (when the sample reached the target temperature); N_res_ is the residual microbial concentration at the starting point of the tail (after stabilization at the end of the decrease); k_max_ is the maximum specific inactivation rate (1/min); t is the time (min). Single inactivation curves were built in this study for each treatment temperature. The values of standard error (SE), root mean square error (RMSE), correlation coefficient (*R*^2^) and adjusted *R*^2^ (*R*^2^_adj_) values of models were provided by the GInaFit adding. For selected temperature, thermal death time values of MAP were calculated: D-values (the time in min required to reduce the microbial population by one log at a selected temperature) were estimated from the thermal inactivation curve using Eq. 3:


(3) $$D-value=\frac{\ln\left(10\right)}{k_{\max}}$$

z-value (the increase of temperature required for 1 log reduction in D-value) were determined from the decimal reduction time curves of log D-value versus temperature and were calculated as z = −slope^−1^. Log survival of MAP during the linear inactivation portion of each thermal curve, was also calculated as log (N/N_0_).

## Results

Control milk resulted negative to MAP (limit of quantification: 1 log CFU/mL), while spiked milk showed a concentration ranging from 6.32 to 6.8 log CFU MAP/mL.

During the cheesemaking, the addition of starter cultures (3.23 ± 0.52 log CFU/mL in milk) and calf rennet caused the milk coagulation and the whey separation within 60 min at 36°C. Subsequently, the curd was matured under whey reaching the pH values of 5.17 ± 0.07, within the range of 5.1–5.3 appropriate for the stretching step. Lactic acid bacteria concentration, pH and aw values reached by the mature curd are reported in Table 2.

**Table 2:** Concentration of Lactic Acid Bacteria (LAB; log CFU/g) and values of pH and aw of milk and curd used for thermal inactivation study.

Parameters	Milk	Curd
LAB	3.23 ± 0.52	5.04 ± 0.47
pH	6.5 ± 0.06	5.17 ± 0.07
aw	Nd	0.99 ± 0.01

Nd, not determined. Data are the average of 2 values per 6 independent experiments

Isothermal treatments of curd vacuum-packed were performed at different temperatures within the range between 60°C and 75°C simulating the stretching conditions of pasta-filata cheese process. The time/temperature profiles registered during all experiments are show in Supplemental Figure S1 (Supplemental File).

When the sample reached the target temperature of 60, 64 and 68°C, the N_0_ of MAP was between 5.61 and 5.82 log CFU/g, while N_0_ resulted from 4.30 to 4.61 log CFU/g in the experiments at 70, 73 and 75°C.

**Figure 2:**
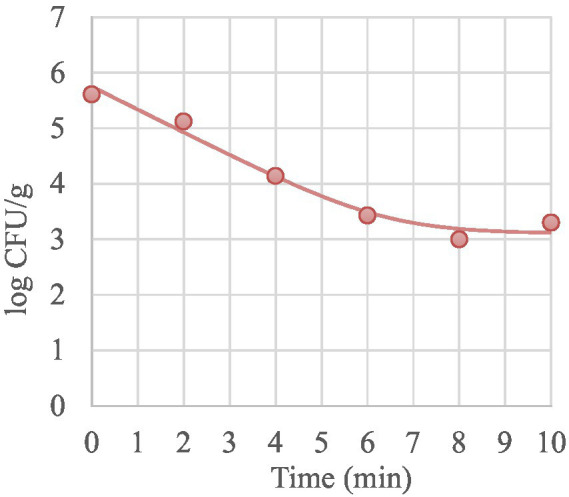
Representative thermal inactivation curve of MAP in mature curd treated at 64°C. Symbols are the plate count of MAP. Line is the curve generated from the “log-linear +Tail” model (Geeraerd et al., 2000).

During the isothermal treatments, MAP inactivation was described by log-linear model or, when the curd was treated at 60°C and 64°C, by biphasic inactivation curve (log-linear + Tail) with a N_res_ up to 3.10 log CFU/g indicating the presence of a resistant subpopulation. This model allows to describe broken curves with different subpopulation resistances by taking into consideration a fraction of the survivors that seem to be more resistant at the lowest temperatures tested in this study. In Figure 2 is shown the representative biphasic curve obtained for MAP at 64°C.

**Figure 3:**
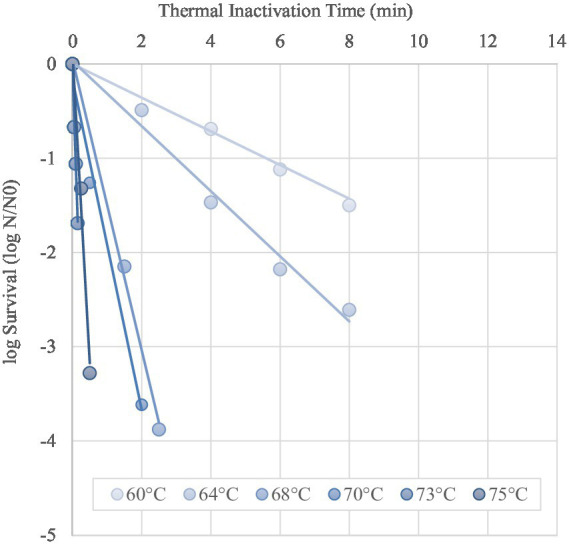
Log Survival curves determined from the log-linear portion of the inactivation curves (solid lines) for MAP (symbols) in curd during the simulation of the stretching step at different temperatures (isothermal treatments). Each curve represents an independent experiment.

**Figure 4:**
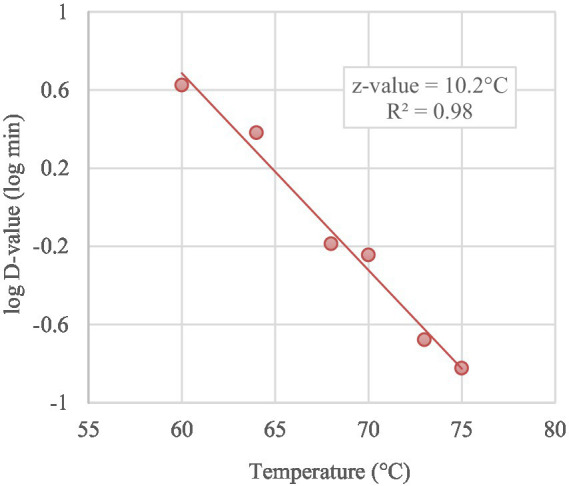
Thermal death time curve (z-value) for MAP in curd treated from 60°C to 75°C obtained by *D*-values in a log scale (symbols) as function of treatment temperatures.

Based on the trend lines of the log-linear model (Figure 3), the K_max_ of MAP ranged from 15.11/min to 0.55/min at 75°C and 60°C respectively, with D-values increasing from 0.15 min (D_75_-value), 0.21 min (D_73_-value), 0.57 min (D_70_-value), 0.65 min (D_68_-value), 2.41 min (D_64_-value) to 4.22 min (D_60_-value), as reported in Table 3. Thermal death time curve (z-values) for the thermal treatments is reported in Figure 4 and the calculated z-value was 10.2°C.

**Table 3:** Thermal inactivation parameters estimated for MAP in curd during isothermal treatments from 60°C to 75°C.

Temperature (°C)	*Kmax* (1/min)	SE	RMSE (Log CFU/g)	*R* ^2^	*R* ^2^_adj_	D-value (min)
60	0.55	0.4	0.64	0.80	0.6	4.22
64	0.96	0.1	0.38	0.98	0.96	2.41
68	3.55	0.2	0.43	0.99	0.99	0.65
70	4.04	0.2	0.47	0.99	0.99	0.57
73	10.76	3.3	1.82	0.68	0.61	0.21
75	15.11	0.7	0.81	0.99	0.99	0.15

kmax, specific inactivation rate; SE, standard error; RSME, root mean squared errors; *R*^2^, regression coefficient; *R*^2^_adj_, adjusted coefficient of determination (as given in GInaFit); D-value: decimal reduction time.

## Discussion

Although the role of MAP in the etiology of some human diseases has been debated since the last century (Chiodini et al., 2012), final conclusions have not been so far achieved.

In general, people could be exposed to MAP by many different sources: consumption of milk, cheeses, meat and water. In the present paper we have focused our attention on the production of cheese, whose consumption represents one of the most accredited way of exposure of people to MAP (Gill et al., 2011). One of the principal reasons why MAP is suspected to represent a risk for human consumers is because this bacterium has revealed to be particularly resistant to heat. Indeed, in some cases, when the starting concentrations were very high, even the pasteurization treatment did not prove sufficient to completely eliminate the bacterium (EFSA, 2017; Mullan, 2019). Concerning pasteurization of milk, inactivation parameters of MAP had been investigated by other authors and reviewed by Mullan (2019) reporting D72°C values ranged from 1.2 to 13 s, z-values ranged from 6.6 to 9.76°C. Otherwise, no information are currently available about the survival and inactivation parameters of MAP during the production process of pasta filata-cheeses, like mozzarella. Data about the survival of MAP during the manufacturing process (but not during isothermal conditions) are currently available for other type of cheeses. Sung and Collins (Sung and Collins, 2000) reported a D-value of 59.9 days for a Hispanic-style soft white cheese, while 27.8 and 45.5 days are the D-values for Switzerland hard and semi-hard cheeses (Swiss Tilsiter; Spahr and Schafroth, 2001), respectively. Cheddar cheese showed D-values ranging from 90 to 107 days (Donaghy et al., 2004). However, these inactivation values were calculated during the cheesemaking or during the ripening of cheese and thus, they can be affected by several dynamic conditions due to changes in temperature, pH, salt concentration and moisture content. In addition, a study conducted on white cheese made by ultra-filtered milk in Iran showed a reduction in MAP starting from day 30 of maturation (Hanifian, 2014). Another study that investigated the inactivation rate of MAP during the production process of Italian hard cheeses (Grana Padano and Parmigiano Reggiano) detected no viable MAP cells from the second (mixture of wild strains) and the third month of ripening (ATCC strain; Cammi et al., 2019). Finally, a recent Italian work on fresh cheese made by milk goat in Piedmont (Italy) did not detected MAP by culture assay at 10 days of ripening (Pagliasso et al., 2021).

Relatively to mozzarella cheese, the stretching phase has been compared to the milk pasteurization (65°C for 30 min), suggesting how the former was more efficient in the reduction of load of some bacteria, such as *Listeria* spp., *Mycobacterium fortuitum* and *Salmonella* typhimurium, and less efficient for others, like *Staphylococcus aureus* (Raimundo et al., 2013). However, the stretching process is not an isothermal treatment, being the temperature reached inside the mass of curd during the working process not homogeneous. On the other hand, the ratio between surface and volume of the curd tends to increase during this process, increasing in this way the exposure to the heat of all microorganisms inside the curd. Finally, it should also be kept in consideration that the stretching phase can change depending on the kind of products and the area of production, making this process very difficult to monitor and standardize (Ricci et al., 2020). Considering the above cited considerations, in order to define the principal kinetic parameters of MAP inactivation during the production of pasta-filata cheese, collected data were fitted with a primary model, which encompassed the reduction of MAP load inside each curd sample submitted to different isothermal treatments. Subsequently, a further model was built in order to predict the variations of MAP survival as a function of temperature changes.

Precise evaluation of the thermal resistance of microorganisms in food processing is critical for the design of thermal processes. According to our experimental study, in recent years, multiple-strains of microorganism cocktails have been used in a preferential way over single individual strains in determining the thermal inactivation kinetics and lethality of pathogens in foods, due to the observed variation in thermal inactivation kinetics among strains (Murphy et al., 2000; Rajkowski, 2012; Juneja et al., 2013; Wang et al., 2017; Ballom et al., 2020; Channaiah et al., 2021; Ferigolo et al., 2021).

Furthermore, for most foodborne pathogens in a food matrix under isothermal conditions, the cell population generally decreases exponentially with heating time and, therefore, can be described by 1st-order kinetics, also for mixed cultures. When biphasic curves are used to describe the pathogen inactivation, they represent the fractions of initial population (major and minor subpopulations) with different heat resistance and specific inactivation rates: the more heat-sensitive microorganisms will be preferentially inactivated, followed by the heat-resistant ones, explaining the “tail” effect observed in some survival curves (Geeraerd et al., 2005; Juneja et al., 2006). The inactivation curves obtained in this study displayed tailing after a log-linear decrease for MAP at 60°C and 64°C, indicating that heating at such temperatures is not efficient to completely inactivate the pathogen. For this reason, selected conditions (temperature and time) that increase treatment lethality is needed to improve the food safety concerning the MAP survival. In fact, when the heating temperature increased to 68°C, 70°C, 73°C and 75°C, the “tail” effect was not observed in our study, suggesting these as lethal temperatures for MAP, and causing exponential decreases during the time.

Regarding the present study, D-values of the portions of curd analyzed from 60°C to 75°C dropped from 4.22 min to 0.15 min. The correlation between D-values and temperature resulted in the building of a secondary model which allowed the calculation of the z-value. According to our study, the z-value of MAP in curd submitted to these isothermal treatments resulted 10.2°C, a value very similar to those reported for *L. monocytogenes* (11.1°C) during the production of curd made from buffalo milk (Ricci et al., 2020). Conversely, for the milk pasteurization process at 71°C and 72°C, z-values ranged from 6.6°C to 9.8°C (Sung and Collins, 2000; Rademaker et al., 2007; Foddai et al., 2010).

In a study carried out on bulk tank milk of 2,934 herds of Emilia Romagna Region (Italy) by Ricchi et al. (2016), the maximum MAP load observed was 1,424 MAP cells/mL, corresponding to 14–142 CFU/ml (1.15–2.15 log CFU/mL; Ricchi et al., 2017). Such data, together with the thermal kinetics of MAP in curd, aid the design of food thermal treatments to achieve the required reduction of a target pathogen in a food product. In fact, in accordance with Stringer and Metris (2018), once measured, z-values can be used to determine time–temperature combinations that give a specified kill of bacteria. For example, if the z value of MAP in curd is 10.2°C, a heat treatment of 2 min at 68°C would reduce 3 log CFU/g to same extent as heating at 57.8°C for 20 min or at 78.2°C for 0.2 min.

Thus, D- and z-values are used for describing the efficacy of static pathogen inactivation treatments and *F*-values can be used for evaluating that of a dynamic process. In practice, heat treatments are usually dynamic, i.e., the temperature varies with time. Assuming that the effect of a heat treatment is cumulative, using an integrated F-value allows the severity of dynamic thermal processes to be calculated. This allows the equivalence of non-isothermal treatments to be calculated and compared (Stringer and Metris, 2018).

Taking into consideration that MAP can not multiply outside the host, our results show how the stretching phase is likely to reduce the load of MAP in a way directly dependent by the time of exposure and temperature of stretching water. It must be underlined that MAP concentrations used to spike the milk were extremely high (5–6 log CFU/ml of milk) if compared to the real contamination levels. In fact, some studies reported how MAP load is low in bulk milk (Okura et al., 2012; Mullan, 2019). However, higher MAP loads due to accidental contamination during milking procedures, especially in herds with MAP super shedders (Vissers et al., 2007), can not be excluded. Thus, considering the high heat resistance of MAP and its potential role in the pathogenesis of human diseases, the knowledge of the kinetic parameters of MAP inactivation in these dairy products is pivotal.

## Conclusion

In this study, predictive models have been used to describe the inactivation of MAP at different temperatures, typically used for the stretching step during the cheesemaking of mozzarella as well as pasta-filata cheeses, and to describe its resistance to the thermal treatments in terms of D- and z-values.

This is the first report on thermal inactivation of MAP in curd for pasta-filata cheese. Data reported in this study can be used to predict the time/temperature combination needed to obtain a certain MAP log reduction during the curd stretching step, helping the processors in optimization or validation processes when raw milk is used to produce pasta-filata cheeses, designing acceptance limits on critical control points to ensure safety against MAP.

Further research is needed to investigate the effects of changing temperatures over time in real dynamic conditions and to determine the cumulative lethality during the cheesemaking of pasta-filata cheese, with the ultimate goal to evaluate the validity of the investigated model directly during the entire cheese production process.

## Data availability statement

The raw data supporting the results and the conclusion in this article are available by the authors, further inquiries can be directed to the corresponding author.

## Author contributions

FB, ED, SR, EC-C, and PM: investigation and methodology. FB, ED, SR, EC-C, and CC: methodology and data analysis. FB, ED, SR, EC-C, PM, and MR: formal analysis, data curation. ED and MR: project administration, supervision, writing –review and editing. MR, NA, CG, and ML: resources and funding acquisition. ED and MR: conceptualization, supervision, and writing –original draft. FB, ED, SR, EC-C, PM, CC, NA, CG, ML, and MR: writing –review and editing. All authors contributed to the article and approved the submitted version.

## Funding

This work was supported by Italian Minister of Health within the Project IZS LER 11/18 RC (CUP E56C18001810001) “Studio sulla sopravvivenza di *Mycobacterium avium* subsp. *paratuberculosis* al processo di produzione della mozzarella ottenuta da latte vaccino e bufalino.”

## Conflict of interest

The authors declare that the research was conducted in the absence of any commercial or financial relationships that could be construed as a potential conflict of interest.

## Publisher’s note

All claims expressed in this article are solely those of the authors and do not necessarily represent those of their affiliated organizations, or those of the publisher, the editors and the reviewers. Any product that may be evaluated in this article, or claim that may be made by its manufacturer, is not guaranteed or endorsed by the publisher.
