# Supplementary material for: Isothermal inactivation of Mycobacterium avium subsp. paratuberculosis in curd simulating the stretching phase in pasta-filata cheese process
Source: Front Microbiol. 2022 Dec 1;13:1052222. doi: 10.3389/fmicb.2022.1052222 (PMC9751633; doi:10.3389/fmicb.2022.1052222)
Supplement: Supplementary file 1 [file Data_Sheet_1.docx]

Supplementary Material

**Supplementary Figure 1.** Temperature profiles of the isothermal curves (▬) and come up times of each experiment (⁃⁃⁃).
